# Alterations in ether lipid metabolism in obesity revealed by systems genomics of multi-omics datasets

**DOI:** 10.1371/journal.pbio.3003349

**Published:** 2025-08-28

**Authors:** Yvette L. Schooneveldt, Sudip Paul, Kevin Huynh, Habtamu B. Beyene, Nat A. Mellett, Gerald F. Watts, Joseph Hung, Jennie Hui, John Beilby, John Blangero, Eric K. Moses, Jonathan E. Shaw, Dianna J. Magliano, Marcus M. Seldin, Brian G. Drew, Anna C. Calkin, Corey Giles, Peter J. Meikle

**Affiliations:** 1 Baker Heart and Diabetes Institute, Melbourne, Victoria, Australia; 2 Faculty of Medicine, Nursing & Health Sciences, Monash University, Melbourne, Victoria, Australia; 3 Baker Department of Cardiovascular Research Translation and Implementation, La Trobe University, Bundoora, Victoria, Australia; 4 Baker Department of Cardiometabolic Health, University of Melbourne, Parkville, Victoria, Australia; 5 School of Medicine, University of Western Australia, Perth, Australia; 6 Lipid Disorders Clinic, Department of Cardiology, Royal Perth Hospital, Perth, Australia; 7 School of Biomedical Sciences, University of Western Australia, Perth, Australia; 8 School of Population and Global Health, University of Western Australia, Crawley, Western Australia, Australia; 9 South Texas Diabetes and Obesity Institute, The University of Texas Rio Grande Valley, Brownsville, Texas, United States of America; 10 School of Biomedical Sciences, University of Western Australia, Crawley, Western Australia, Australia; 11 Menzies Institute for Medical Research, University of Tasmania, Hobart, Tasmania, Australia; 12 School of Public Health and Preventative Medicine, Monash University, Melbourne, Victoria, Australia; 13 Department of Biological Chemistry Centre for Epigenetics and Metabolism, University of California, Irvine, California, United States of America; The Francis Crick Institute, UNITED KINGDOM OF GREAT BRITAIN AND NORTHERN IRELAND

## Abstract

Ratios between two metabolites are sensitive indicators of metabolic changes. Lipidomic profiling studies have revealed that plasma ether lipids, a class of glycero- and glycerophospho-lipids with reported health benefits, are negatively associated with obesity. Here, we utilized lipid ratios as surrogate markers of lipid metabolism to explore the processes underlying the inverse relationship between ether lipid metabolism and obesity. Plasma lipidomics data from two independent human cohorts (*n* = 10,339 and *n* = 4,492) were integrated to assess the associations between 82 lipid ratios and obesity-related markers in males and females. Results were externally validated using mouse transcriptomics data from the Hybrid Mouse Diversity Panel (*n* = 152−227 across 74 strains). Genome-wide association studies using imputed genotypes from a population cohort (*n* = 4,492) were performed to examine the genetic architecture of the ratios. Findings showed that waist circumference (WC), body mass index, and waist–hip ratio were inversely associated with total plasmalogens relative to total phospholipids in both sexes. Ratios comprising product–substrate pairs positioned either side of enzymes involved in plasmalogen synthesis and degradation showed positive and negative associations with WC, respectively. Branched-chain fatty acids negatively correlated with WC, while omega-6 polyunsaturated fatty acids exhibited differing associations depending on their position within the pathway. Mouse transcriptomics corroborated these results. Genomics data showed strong associations between ratios containing choline-plasmalogens and single-nucleotide polymorphisms in the transmembrane protein 229B (*TMEM229B*) gene region. This work demonstrates the utility of lipid ratios in understanding lipid metabolism. By applying the ratios to multi-omic datasets, we identified alterations in enzymatic activity and genetic variants likely affecting ether lipid synthesis in obesity that could not have been obtained from lipidomics data alone. Additionally, we characterized a potential role for *TMEM229B*, offering new perspectives on ether lipid metabolism and regulation.

## Abstract 2

Lipidomic profiling studies have linked plasma ether lipids with obesity. This study analyzes a set of biologically informed lipid ratios to further explore functional shifts in ether lipid metabolism, revealing obesity-related alterations in enzymatic activity and genetic variants that are not apparent in traditional lipidomic analyses.

## Introduction

Lipids are diverse biomolecules essential for human life. They are critical components of cellular membranes and orchestrate numerous functions, including intracellular signaling, protein trafficking, energy storage, and hormone production [1]. Lipids have a high degree of specialization and are classified based on their structural and biological properties. Advances in the field of lipidomics have helped define complex relationships between lipid dysregulation and obesity. Of note, several lipidomic profiling studies have revealed that a class of lipids, termed ether lipids, are negatively associated with hypertension, type 2 diabetes (T2D), metabolic dysfunction-associated steatotic liver disease, and obesity [2–5].

Ether lipids encompass glycero- and glycerophospho-lipids that are characterized by an ether or vinyl-ether linked fatty alcohol at the *sn-1* position on a glycerol backbone. This is contrary to conventional glycerol-based lipids, where fatty acids are attached exclusively through ester bonds. Approximately 20% of the total phospholipid pool in mammalian cells have an ether-linked moiety, although their relative abundance varies between tissue types [6]. Studies have shown that ether lipids are critical components of subcellular membranes, giving rise to their functional roles in regulating and maintaining membrane homeostasis [7]. Plasmalogens are the most abundant class of ether lipids that are defined by a vinyl-ether linkage at the *sn-1* position. This linkage gives rise to their potent antioxidant properties, while they have also been reported to facilitate mitochondrial fission and fusion events, cholesterol transport, immune cell signaling, and preserve membrane dynamics [8–14].

Previously, we comprehensively examined the relationship between 577 lipid species with age, sex, and body mass index (BMI) in two large population cohorts [15]. The study reported a significant negative correlation between BMI and multiple plasmalogen species [15]. This work aligned with findings from Pietiläinen *and colleagues*, who reported a significant reduction in ether lipid concentrations in obese twins [2]. It is proposed that this decrease in plasmalogens may explain, in part, the elevated oxidative stress and inflammation commonly associated with obesity, given their antioxidant and anti-inflammatory actions [16]. However, it remains unclear whether this reduction stems from their susceptibility to oxidation or a dysregulation of their metabolism. Furthermore, plasmalogens are known to regulate energy metabolism, and their dysregulation may contribute to the disruption of energy homeostasis that is commonly observed in obesity [17].

Ratios between specific metabolites, including lipids, have been used as informative markers of disease onset and progression, as well as indicators of biological functions, such as enzymatic activity [18–22]. This study aimed to generate ratios between specific lipid pairs to capture key features of ether lipid metabolism and investigate their association with obesity. For example, a ratio between lipids that are a precursor and product of a single enzyme may reflect the activity of that enzyme [22]. Alternatively, a ratio between lipids that are positioned at the start and end of a particular pathway may indicate changes throughout that pathway [23]. By integrating these ratios with lipidomic, transcriptomic, and genomic data, we sought to examine their associations with obesity-related measures to uncover the potential molecular drivers underpinning ether lipid dysregulation in obesity.

## Methods

### Study cohorts

#### The Australian, Diabetes, Obesity and Lifestyle Study.

The Australian, Diabetes, Obesity and Lifestyle Study (AusDiab) is a large cross-sectional population-based study that commenced in 1999–2000 with a total of 11,247 participants aged ≥25 years. The study recorded the prevalence and incidence of diabetes, obesity, hypertension, and kidney disease within Australia. Detailed descriptions on the study population and clinical measurements, including fasting blood glucose, total cholesterol, waist circumference (WC), waist–hip ratio (WHR), BMI, and behavioral risk factors are described elsewhere [24]. A summary of the descriptive statistics can be found in S1 Table. This study utilized plasma lipidomics data from a total of 10,339 participants at baseline.

#### Busselton Health Study.

The Busselton Health Study (BHS) is an ongoing community-based study that commenced in 1966 in the town of Busselton, Western Australia. The study explores health status, lifestyle factors, and environmental exposures of individuals within the region. Clinical measurements are described in S1 Table, while details on the study participants have been published previously [25]. In this study, lipidomics data and imputed genotypes from a total of 4,492 participants from the 1994 to 1995 recall were used [26].

#### Ethics statement.

This study used datasets from the AusDiab biobank and BHS. The AusDiab study was approved by the Alfred Human Research Ethics Committee, Melbourne, Australia (project approval number; 41/18) and the BHS cohort was approved by the University of Western Australia (UWA) Human Research Ethics Committee (HREC) (approval numbers; 608/15 and RA/4/1/7894) and the Western Australian Department of Health HREC (RGS03656). Both studies were conducted in accordance with the ethical principles of the Declaration of Helsinki and informed consent was obtained from all participants.

### Hybrid Mouse Diversity Panel

The Hybrid Mouse Diversity Panel (HMDP) was developed to establish a panel of isogenic mouse strains that capture genetic variations similar to that found in natural populations. Details on the extensive phenotyping and multi-omic analyses across disease models are available elsewhere [27–29]. To explore the effects of a high-fat/high-sugar (HFHS) diet on ether lipid metabolism, the study matched 74 well-characterized inbred mouse strains (2–3 mice per strain) from two HMDP cohorts. This generated a comprehensive dataset comprising liver microarray data from 8-week-old male mice fed either a standard chow (*n* = 152) or HFHS diet (*n* = 222) for 8 weeks [30,31]. The differences in sample size reflect the original study designs, as the HFHS cohort included more mice per strain. To assess sex differences, liver microarray data from 108 inbred male and female mouse strains (2–3 mice per strain) were used [31]. At 8 weeks, male (*n* = 227) and female (*n* = 206) mice were fed a HFHS diet for 8 weeks. All microarray data used in this study are publicly available from Gene Expression Omnibus under the accession codes: GSE16780 and GSE64769 [30,31].

### Lipidomic profiling

Extensive details on the lipidomic profiling of the AusDiab and BHS cohorts have been published previously [15,32]. Briefly, 10 μL of fasting plasma (AusDiab) or fasting serum (BHS) was mixed with 100 μL butanol/methanol (1:1 v/v) plus 10 mM ammonium formate and a mix of 23 internal standards (ISTD). Targeted lipidomic profiling was performed on the crude lipid extracts using liquid chromatography electrospray ionization-tandem mass spectrometry (LC–MS/MS) on an Agilent 6490 QqQ mass spectrometer with an Agilent 1290 series high-performance liquid chromatography (HPLC) system. Details on the standardized method and chromatography gradient have been described elsewhere [33,34].

### Quantification of lipid species

Results from the chromatographic data was analyzed using Mass Hunter Quant v10.0 where peaks were integrated and assigned to a specific lipid species based on dynamic scheduled multiple reaction monitoring (dMRM) ion pairs and retention time. The exact dMRM transitions for the datasets can be viewed in S2 Table. Relative lipid abundances were calculated by relating the area under the curve for each analyte to the corresponding ISTD and multiplying by the amount of ISTD added into the sample. In-house pipelines were used for quality control and filtering of lipid concentrations. An imputation technique was applied to capture unmeasured high-resolution lipid species in the BHS dataset using a reference cohort [35]. The abundance of lipid classes was calculated from the sum of individual species within said class. In total, 735 lipid species from 39 classes were quantified in the AusDiab and BHS cohorts.

### Statistical analysis

Lipid concentrations between the AusDiab and BHS cohorts were standardized to correct for misalignments between the datasets. For the AusDiab cohort, a correction factor was calculated by determining the ratio of the median NIST-SRM-1950 concentration for each lipid species measured in the dataset to the consensus NIST-SRM-1950 concentration (based on the analysis of the NIST-SRM-1950 reference plasma on 12 separate occasions within our laboratory). Since the BHS cohort did not include NIST-SRM-1950 samples, an alternative approach was used. Sub-cohorts (*n* ~ 1,000) within the BHS and AusDiab datasets were created by matching participants based on age, sex, BMI, total cholesterol, and disease prevalence. For each lipid, a correction factor was calculated as the ratio of median lipid concentrations between the matched AusDiab and BHS sub-cohorts, and applied to the BHS data. This approach ensured that BHS lipid concentrations were aligned with those from the AusDiab in the absence of NIST-SRM-1950 data. Within the cohorts themselves, pooled quality control (PQC) samples were interspersed throughout the dataset to monitor and correct for batch effects. Median centering was applied to the PQC samples to minimize technical variation and ensure consistency across the batches.

Lipid ratios were generated from known biological pathways and formulated by dividing the concentration of a particular lipid class or species by the concentration of another class or species. Each lipid pair was directly related, either as product-substrates of enzymatic conversions or the initial and final components of a lipid metabolic pathway. The complete list of ratios, which aimed to capture changes in ether lipid composition (acyl- and alkenyl-chains), enzymatic activity, and central pathways involved in ether lipid and fatty acid synthesis, are provided in S3 Table. A total of 82 lipid ratios were generated using plasma lipidomic data from the AusDiab and BHS cohorts. Linear regression analysis, adjusting for age and sex, was used to assess the relationship between each ratio and measures of obesity, including WC, BMI, and WHR. To explore sex-specific associations, the same linear models were fitted with an interaction term for sex. Lipid ratios were log-transformed and scaled to zero mean and one-unit standard deviation. *p-gains*, representing the increase in association strength from using the lipid ratio, were calculated by dividing the lower *p*-*value* of the lipid species in the ratio by the *p-value* of the lipid ratio itself [36]. *p-gain* values were deemed significant if they exceeded 10 × the number of ratios tested (*p-gain* >820), as outlined by Petersen and colleagues [36]. All association *p-values* were corrected for multiple comparisons using the false discovery rate method of Benjamini and Hochberg [37].

### Lipid set enrichment analysis

Lipid set enrichment analysis (LSEA) was performed on associations with WC in the AusDiab cohort to identify enrichment of lipid sets across the entire lipidome, while accounting for the correlation between lipid species. Lipid sets were defined based on structural and ontological criteria, representing lipid classes, subclasses, and features. The association t-statistic was recorded for each lipid species against WC, adjusting for age and sex. Covariate-adjusted lipid correlations were calculated using residuals of lipid species after regressing out covariates. Enrichment scores were generated by summing the t-statistic for all lipids in a set and adjusting for the correlation (taking as the denominator the square root of the sum of the adjusted correlation matrix specific to the lipid set), yielding a score that reflects the degree of enrichment for that lipid set against markers of obesity [38]. Under the null hypothesis, the squared enrichment score tends a chi-squared distribution with large sample sizes, allowing *p-values* to be approximated. *p-values* were corrected for multiple comparisons using the false discovery rate method of Benjamini and Hochberg [37].

### Genome-wide-association statistical analysis

Genotyping for the BHS cohort was performed using the Illumina Human 610K Quad-Bead Chip (*n* = 1,468) and the Illumina 660W Quad Array Bead Chip (*n* = 3,428), as previously described [26,32]. Quality control steps were implemented to ensure high-quality genotype data; individuals with missing genotype data (>3%), sex mismatches, duplicate samples, missing phenotype information, extreme levels of heterozygosity (>5 standard deviations from the mean) or non-European ancestry, were excluded from the analysis [26,32]. For single-nucleotide polymorphisms (SNPs), exclusion criteria included call rates less than 95%, minor allele count less than 10, significant deviations from Hardy-Weinberg equilibrium (*p-value* < 5.0 × 10^−^⁴), mismatches with the Haplotype Reference Consortium reference panel, palindromic SNPs with a minor allele frequency (MAF) greater than 0.4 and those with a MAF difference greater than 0.2 when compared to the HRC panel. Genotype data were subsequently imputed using the HRC reference panel through the Michigan Imputation Server. Following imputation, variants with a minor allele count less than 5 or imputation quality lower than 0.3 were excluded, resulting in 13,887,524 SNPs available for analysis [26,32].

Genome-wide association analysis was performed on lipid ratios using imputed genotype data from the BHS cohort. Lipid ratios were adjusted for age, sex, age^2^, age*sex, age^2^*sex, and the first 10 genomic principal components. Residuals were rank-based inverse normal transformed and analyzed using linear mixed models with an empirical genetic related matrix (GRM) as the random effects (GEMMA v0.98.5) [26,39]. To avoid proximal contamination, the analysis was performed by excluding one chromosome at a time in the GRM calculation. A genome-wide significance threshold of **p-value* *< 5 × 10^−8^ was used to identify SNPs associated with lipid ratios.

## Results

### Lipid ratios explore ether lipid dysregulation in obesity

**Fig 1:**
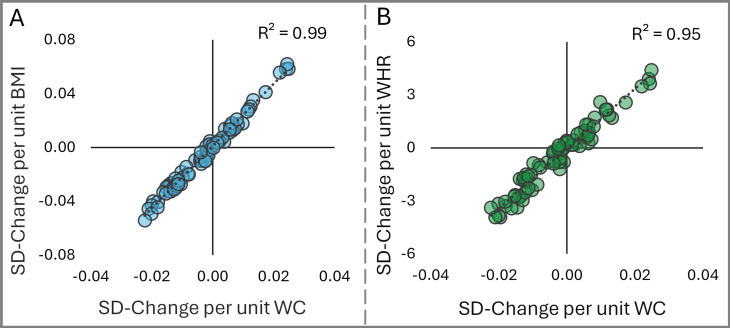
Validation of lipid ratios across markers of obesity. Correlation between the regression coefficients of each lipid ratio in the AusDiab cohort (*n* = 10,399); **A**) waist circumference (*x* axis) and body-mass-index (*y* axis) and **B**) waist circumference (*x* axis) and waist–hip ratio (*y* axis). Lipid ratios were log_2_ transformed, mean-centered, and scaled to standard deviation (SD). WC, waist circumference; BMI, body-mass index; WHR, waist–hip ratio; SD-Change, standard deviation-change.

We generated 82 lipid ratios, drawing on our understanding of lipid metabolism, to explore ether lipid synthesis in the setting of obesity. To account for the inherent limitations of measuring obesity, the study evaluated the associations of each ratio with WC, BMI, and WHR in the AusDiab dataset. Results showed strong correlations between all three measurements (*r*^2^ = 0.99 between WC and BMI, *r*^2^ = 0.96 between WC and WHR) and WC was therefore implemented as the primary outcome for the analysis (Fig 1A and 1B). Results for BMI and WHR can be viewed in S4 Table.

Of the 82 ratios, 68 exhibited significant associations (BH *p-value* <0.05) with WC in the AusDiab dataset (S4 Table). Of these, 34 ratios had a significant *p-gain,* illustrating the ability of the ratios to capture new biological information. At a class level, WC was inversely associated with ethanolamine ether lipids, including alkyl- and alkenyl-phosphatidylethanolamine (PE(O) and PE(P)) classes, relative to ethanolamine phospholipids (PE) (Table 1). This pattern held true across most ratios containing ether lipid classes, namely Total PE Ether/PE, Total PC Ether/PC, PE(P)/PE, PE(O)/PE, PC(P)/PC and PC(O)/PC, with TG(O)/TG as the only exception. Ratios involving lipid classes on either side of an enzyme, such as PE(O)/PE(P), where PE(O) is converted into PE(P) via plasmanylethanolamine desaturase 1 (PEDS1), showed a negative association with WC. Alternatively, the LPE(P)/PE(P) and LPC(P)/PC(P) ratios, which include substrates and products of calcium-independent phospholipase A2 (iPLA2) and lyso-phospholipid acyltransferase (LPAT), displayed positive and negative associations with WC, respectively (Table 1). While these observations were consistent across ratios containing both phospholipid and ether lipid classes (LPE(P)/PE(P), LPC(P)/PC(P), LPE/PE and LPC/PC), only ratios with a phospholipid class in the denominator (LEP/PE and LPC/PC) yielded a significant *p-gain*. This indicates that including ether lipids in the ratio did not strengthen the associations with WC beyond that of the individual lipid classes.

**Table 1:** Associations between ratios with ether lipid classes and waist circumference^1^.

Lipid Class Ratio^2^	SD-change per unit WC [Lipids]	*p-value* [Lipids]	SD-change per unit WC [Ratio]	*p-value* [Ratio]	*p-gain* [Ratio]
Total PE Ether	−0.003	1.05 × 10^−03^	−0.006	7.39 × 10^−13^	<1.00
PE	0.010	3.36 × 10^−35^			
Total PC Ether	−0.015	7.78 × 10^−75^	−0.015	3.94 × 10^−77^	1.98 × 10^02^
PC	0.005	1.38 × 10^−09^			
PE(P)	−0.002	3.05 × 10^−03^	−0.010	4.41 × 10^−39^	7.60 × 10^03^
PE	0.010	3.36 × 10^−35^			
PE(O)	−0.008	4.31 × 10^−22^	−0.014	4.30 × 10^−68^	7.80 × 10^32^
PE	0.010	3.36 × 10^−35^			
PC(P)	−0.015	3.48 × 10^−78^	−0.020	1.09 × 10^−133^	3.20 × 10^55^
PC	0.005	1.38 × 10^−09^			
PC(O)	−0.011	1.48 × 10^−45^	−0.014	4.21 × 10^−73^	3.50 × 10^27^
PC	0.005	1.38 × 10^−09^			
TG(O)	0.013	3.08 × 10^−63^	−0.015	6.07 × 10^−82^	<1.00
TG	0.028	4.59 × 10^−290^			
PE(O)	−0.008	4.31 × 10^−22^	−0.008	1.29 × 10^−25^	3.34 × 10^03^
PE(P)	−0.002	3.05 × 10^−03^			
PC(P)	−0.015	3.48 × 10^−78^	−0.013	3.21 × 10^−59^	<1.00
PE(P)	−0.002	3.05 × 10^−03^			
LPE(P)	−0.005	7.78 × 10^−12^	−0.004	9.61 × 10^−06^	<1.00
PE(P)	−0.002	3.05 × 10^−03^			
LPC(P)	−0.014	1.46 × 10^−66^	−0.001	3.22 × 10^−01^	<1.00
PC(P)	−0.015	3.48 × 10^−78^			
LPE	−0.009	3.98 × 10^−28^	−0.018	2.38 × 10^−123^	1.41 × 10^88^
PE	0.010	3.36 × 10^−35^			
LPC	−0.007	2.62 × 10^−16^	−0.010	2.25 × 10^−37^	1.17 × 10^21^
PC	0.005	1.38 × 10^−09^			

^1^Linear regression analysis, adjusting for age and sex, between lipid ratios and waist circumference in the AusDiab cohort (n = 10,399).

^2^Lipid Ratios read as the first lipid class divided by the second within each box. Lipid ratios were log_2_ transformed, mean centered and scaled to standard deviation. *p-values* were corrected for multiple comparisons using the false discovery rate method of Benjamini and Hochberg. *p-gains* were calculated by dividing the lower *p-value* of the two lipid classes in the ratio by the *p-value* of the lipid ratio. *p-gains* >820 were deemed statistically significant.

SD-change, standard-deviation change; BH *p-value*, corrected *p-value*; PE, phosphatidylethanolamine; PE(O), alkyl-phosphatidylethanolamine; PE(P), alkenyl-phosphatidylethanolamine; Total PE Ether, sum of PE(O) and PE(P) classes; PC, phosphatidylcholine; PC(O), alkyl-phosphatidylcholine; PC(P), alkenyl-phosphatidylcholine; Total PC Ether, sum of PC(O) and PC(P) classes; TG, triacylglycerol; TG(O), mono-alkyl-diacylglycerol; LPE, lyso-phosphatidylethanolamine; LPE(P):, lyso-alkenyl-phosphatidylethanolamine; LPC, lyso-phosphatidylcholine; LPC(P), lyso-alkenyl-phosphatidylcholine.

Ratios between lipid species were used to assess the association between WC and differing acyl- and alkenyl-chains. Alterations in PE(P-16:0/18:2)/PE(P-16:0/20:4) and PE(P-18:0/18:2)/PE(P-18:0/20:4), which contain differing omega-6 (n-6) polyunsaturated fatty acid (PUFA) acyl-chains, revealed arachidonic acid (AA, 20:4) was elevated relative to its precursor linoleic acid (LA, 18:2) (Table 2). This observation changed for ratios containing species from other regions of the pathway, as PE(P-16:0/22:4)/PE(P-16:0/22:5) and PE(P-18:0/22:4)/PE(P-18:0/22:5) demonstrated a reduction in docosapentaenoic acid (DPA, 22:5) relative to docosatetraenoic acid (22:4). Ratios detailing the omega-3 (n-3) pathway, namely PE(P-16:0/18:3)/PE(P-16:0/20:5), PE(P-18:0/18:3)/PE(P-18:0/20:5), PE(P-16:0/20:5)/PE(P-16:0/22:5), PE(P-18:0/20:5)/PE(P-18:0/22:5), PE(P16:0/22:5)/PE(P-16:0/22:6) and PE(P-18:0/22:5)/PE(P-18:0/22:6), did not present with significant *p-gains* despite showing similar associations to the n-6 ratios with increasing WC. Ratios containing plasmalogen species with chimyl alcohol (O-16:0) at the *sn-1* position, including PE(P-16:0/18:2)/PE(P-18:0/18:2), PE(P-16:0/20:4)/PE(P-18:0/20:4), PE(P-16:0/22:4)/PE(P-18:0/22:4) and PE(P-16:0/22:6)/PE(P-18:0/22:6), illustrated a positive association with increasing WC, relative to its downstream product of batyl alcohol (O-18:0) (Table 2).

**Table 2:** Associations between ratios with ether lipid species and waist circumference^1^^.^

Lipid Class Ratio^2^	SD-change per unit WC [Lipids]	*p-value* [Lipids]	SD-change per unit WC [Ratio]	*p-value* [Ratio]	*p-gain* [Ratio]
PE(P-16:0/18:3) [n-3]	−0.003	7.65 × 10^−04^	−0.004	3.04 × 10^−06^	2.52 × 10^02^
PE(P-16:0/20:5) [n-3]	0.001	8.26 × 10^−02^			
PE(P-18:0/18:3) [n-3]	−0.010	1.59 × 10^−34^	−0.002	8.15 × 10^−03^	<1.00
PE(P-18:0/20:5) [n-3]	−0.005	5.82 × 10^−09^			
PE(P-16:0/20:5) [n-3]	0.001	8.26 × 10^−02^	−0.0001	9.30 × 10^−01^	<1.00
PE(P-16:0/22:5) [n-3]	0.003	1.61 × 10^−03^			
PE(P-18:0/20:5) [n-3]	−0.005	5.82 × 10^−09^	−0.004	7.73 × 10^−07^	<1.00
PE(P-18:0/22:5) [n-3]	−0.002	4.15 × 10^−03^			
PE(P-16:0/22:5) [n-3]	0.003	1.61 × 10^−03^	0.002	4.43 × 10^−03^	<1.00
PE(P-16:0/22:6) [n-3]	0.000	9.68 × 10^−01^			
PE(P-18:0/22:5) [n-3]	−0.002	4.15 × 10^−03^	0.006	1.99 × 10^−16^	<1.00
PE(P-18:0/22:6) [n-3]	−0.009	1.59 × 10^−31^			
PE(P-16:0/18:2) [n-6]	−0.004	8.99 × 10^−07^	−0.012	7.31 × 10^−52^	5.95 × 10^36^
PE(P-16:0/20:4) [n-6]	0.006	4.35 × 10^−15^			
PE(P-18:0/18:2) [n-6]	−0.010	2.13 × 10^−34^	−0.011	7.25 × 10^−39^	2.94 × 10^04^
PE(P-18:0/20:4) [n-6]	0.000	5.98 × 10^−01^			
PE(P-16:0/20:4) [n-6]	0.006	4.35 × 10^−15^	0.004	4.86 × 10^−07^	<1.00
PE(P-16:0/22:4) [n-6]	0.004	6.24 × 10^−08^			
PE(P-18:0/20:4) [n-6]	−0.0004	5.98 × 10^−01^	−0.001	2.15 × 10^−01^	2.52 × 10^ + 00^
PE(P-18:0/22:4) [n-6]	0.001	4.41 × 10^−01^			
PE(P-16:0/22:4) [n-6]	0.004	6.24 × 10^−08^	0.006	4.15 × 10^−15^	1.50 × 10^07^
PE(P-16:0/22:5) [n-6]	−0.002	2.05 × 10^−02^			
PE(P-18:0/22:4) [n-6]	0.001	4.41 × 10^−01^	0.008	3.92 × 10^−26^	6.37 × 10^09^
PE(P-18:0/22:5) [n-6]	−0.007	2.50 × 10^−16^			
PE(P-16:0/18:2)	−0.004	8.99 × 10^−07^	0.013	2.81 × 10^−61^	7.58 × 10^26^
PE(P-18:0/18:2)	−0.010	2.13 × 10^−34^			
PE(P-16:0/20:4)	0.006	4.35 × 10^−15^	0.012	1.32 × 10^−53^	3.28 × 10^38^
PE(P-18:0/20:4)	0.000	5.98 × 10^−01^			
PE(P-16:0/22:4)	0.004	6.24 × 10^−08^	0.006	6.80 × 10^−13^	9.18 × 10^04^
PE(P-18:0/22:4)	0.001	4.41 × 10^−01^			
PE(P-16:0/22:6)	0.000	9.68 × 10^−01^	0.017	4.8 × 10^−105^	3.32 × 10^73^
PE(P-18:0/22:6)	−0.009	1.59 × 10^−31^			

^1^Linear regression analysis, adjusting for age and sex, between lipid ratios and waist circumference in the AusDiab cohort (*n* = 10,399).

^2^Lipid Ratios read as the first lipid species divided by the second within each box. Lipid ratios were log_2_ transformed, mean centered and scaled to standard deviation. *p-values* were corrected for multiple comparisons using the false discovery rate method of Benjamini and Hochberg. *p-gains* were calculated by dividing the lower *p-value* of the two lipid classes in the ratio by the *p-value* of the lipid ratio. *p-gains* >820 were deemed statistically significant.

SD-change, standard-deviation change; BH *p-value*, corrected *p-value*; n-3, omega-3 pathway; n-6, omega-6 pathway; PE(P), alkenyl-phosphatidylethanolamine; −16:0, chimyl alcohol; −18:0, batyl alcohol; 18:2, linoleic acid; 18:3, -linoleic acid; 20:4, arachidonic acid; 20:5, eicosapentaenoic acid; 22:4, adrenic acid; 22:5 (n-3 and n-6), docosapentaenoic acid; 22:6, docosahexaenoic acid; 24:4, tetracosatetraenoic acid; 24:5, tetracosapentaenoic acid; 24:6, tetracosahexaenoic acid.

To further validate the findings, we repeated our analysis using an independent dataset (S5 Table). Results showed 68 of the total 82 ratios had consistent associations with WC between the original AusDiab and external BHS cohorts (S1 Fig). 9 ratios exhibited opposing associations with WC, namely LPC/PC, LPC(O)/PC(O), LPE(P)/PE(P), LPC(20:4) [sn2]/LPC(22:4) [sn2], PE(P-18:0)/20:5)/PE(P-18:0/22:5) [n3], PE(P-16:0)/18:3:5)/PE(P-18:1/18:3), PE(16:0_18:2)/PE(16:0_20:4), LPC(22:5) [sn2]/LPC(22:6) [sn2] and PE(P-16:0/22:5) [n3]/PE(P-16:0/22:6). These inverse correlations are likely driven by differences in sample type and underlying cohort characteristics.

### Lipid ratios demonstrate sex interactions between ether lipid synthesis and markers of obesity

**Fig 2:**
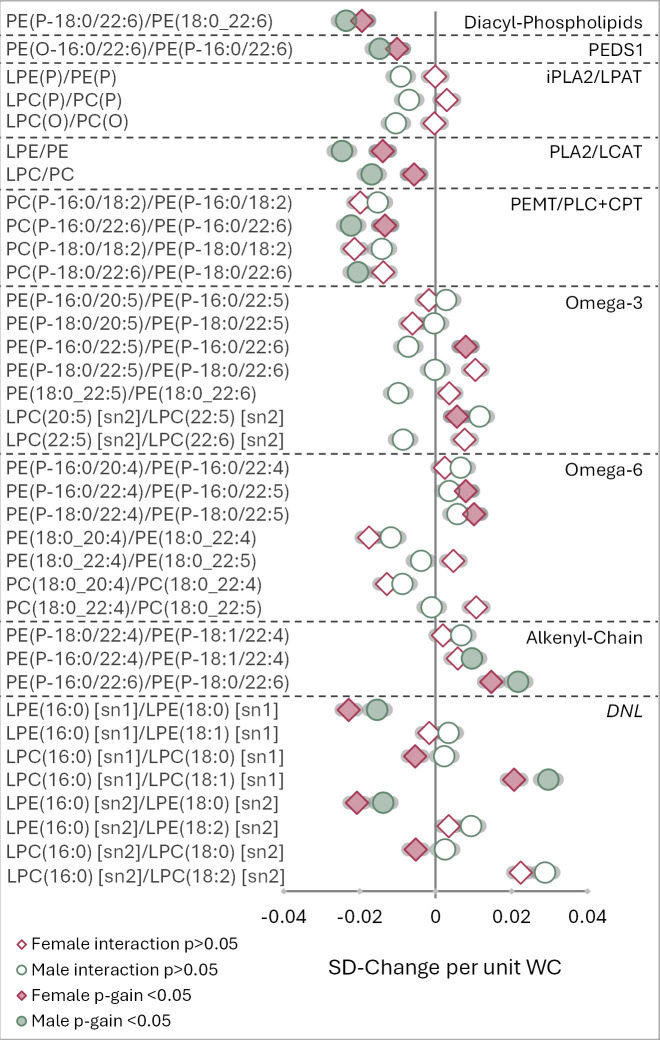
Sex-specific associations between 82 lipid ratios and waist circumference. Linear regression analysis, including sex as the interaction term and adjusting for age, was performed between 82 lipid ratios and waist circumference (WC) in the AusDiab cohort (*n* = 10,399). Hollow points (green circles = male; pink diamonds = female) depict ratios with significantly different associations with WC between males and females (*interaction p-value* <0.05). Ratios with significant *p-gains* (*p-gain* >820) are overlayed in coloured points (green circles = male; pink diamonds = female). Lipid ratios were log_2_ transformed, mean-centered and scaled to standard deviation. *p-values* were corrected for multiple comparisons using the false discovery rate method of Benjamini and Hochberg. *p-gains* were calculated separately for males and females by dividing the lower *p-value* of the two lipid classes in the ratio by the *p-value* of the lipid ratio. SD-Change per unit WC: standard deviation-change per unit of WC; *PEDS1*: plasmanylethanolamine desaturase; *iPLA2*: calcium-independent phospholipase A2; *LPAT*: lyso-phospholipid acyltransferase; *PEMT*: phosphatidylethanolamine N-methyltransferase; *PLC*: phospholipase C; *CPT*: choline phosphotransferase; omega-3: omega-3 poly-unsaturated pathway; omega-6: omega-6 poly-unsaturated pathway; *DNL*: *de novo* lipogenesis pathway; PE: phosphatidylethanolamine; PE(O): alkyl-phosphatidylethanolamine; PE(P): alkenyl-phosphatidylethanolamine; LPE: lyso-phosphatidylethanolamine; LPE(P): lyso-alkenyl-phosphatidylethanolamine; PC: phosphatidylcholine; PC(O): alkyl-phosphatidylcholine; PC(P): alkenyl-phosphatidylcholine; LPC: lyso-phosphatidylcholine; LPC(O): lyso-alkyl-phosphatidylcholine; LPC(P): lyso-alkenyl-phosphatidylcholine; 16:0 *sn-1*: chimyl alcohol; 18:0 *sn-1*: batyl alcohol; 16:0 *sn-2*: palmitic acid; 18:0 *sn-2*: steric acid; 18:1 *sn-2*: oleic acid; 18:2 *sn-2*: linoleic acid; 20:4 *sn-2*: arachidonic acid; 20:5 *sn-2*: eicosapentaenoic acid; 22:4 *sn-2*: adrenic acid; 22:5 *sn-2*: docosapentaenoic acid; 22:6 *sn-2*: docosahexaenoic acid; *sn-1*: fatty alkenyl- or acyl-chain located in the *sn-1* position; *sn-2*, fatty acyl-chain located in the *sn-2* position.

Next, we added sex as an interaction term to evaluate sex-specific associations between each lipid ratio and obesity. Results for BMI (S6 Table) and WHR (S7 Table) were consistent with WC. Thirty-six ratios displayed significantly different associations with WC between males and females (interaction *p-value* <0.05). These ratios contained species spanning multiple stages of the ether lipid pathway, indicating that sex has a notable influence on their metabolism. Overall, males exhibited stronger positive and negative correlations between most ratios and WC (20 ratios), though these were not always accompanied by a significant *p-gain*. Instead, most ratios with a *p-gain >820* (15 out of 17) correlated with WC in females. Ratios containing lyso-phospholipids (LPE(16:0) [sn1]/LPE(18:0) [sn1], LPE(16:0) [sn1]/LPE(18:1) [sn1], LPC(16:0) [sn1]/LPC(18:0) [sn1], LPC(16:0) [sn1]/LPC(18:1) [sn1], LPE(16:0) [sn2]/LPE(18:0) [sn2], LPE(16:0) [sn2]/LPE(18:2) [sn2], LPC(16:0) [sn2]/LPC(18:0) [sn2] and LPC(16:0) [sn2]/LPC(18:2) [sn2]) typically showed stronger negative associations with WC in females and stronger positive associations with WC in males (Fig 2). Additionally, ratios containing DPA, such as PE(P16:0/20:5)/PE(P-16:0/22:5), PE(P-16:0/22:5)/PE(P-16:0/22:6), PE(P-18:0/22:5)/PE(P-18:0/22:6), PE(18:0_22:4)/PE(18:0_22:5), PE(18:0_22:5)/PE(18:0/22:6), PC(18:0_22:4)/PC(18:0_22:5) and LPC(22:5)/LPC(22:6), demonstrated opposing associations between sexes (positive in females, negative in males).

### Lipid-set-enrichment analysis explores lipid remodeling associated with markers of obesity

**Fig 3:**
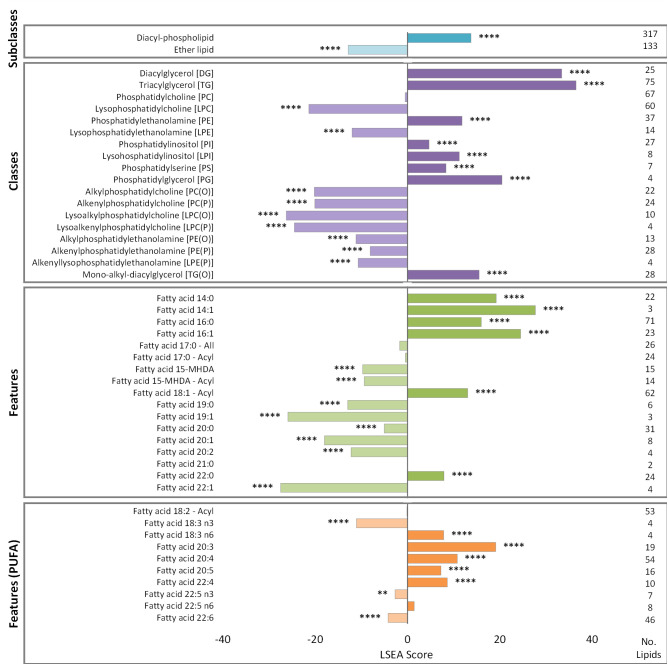
Lipid set enrichment analysis of waist circumference with 735 lipid species. Lipidomic analysis was performed on the AusDiab cohort (*n* = 10,399). Enrichment scores were calculated by summing the association t-statistics for individual lipids against waist circumference. Annotations depict corrected *p-values*. Lipid categories denote a pre-defined lipid set used in the analysis; subclasses are clustered by unique structural features; classes map to lipid species containing the relevant headgroup; features include lipid species containing the respective fatty acid at the *sn-1*, *sn-2* or *sn-3* position; features (PUFA) map to lipid species containing either an omega-3 or omega-6 fatty acid at the *sn-1*, *sn-2* or *sn-3* position; no. lipids details the number of individual lipid species within each lipid set. Lipidomics data was log_2_ transformed and *p-values* were corrected for multiple comparisons using the false discovery rate method of Benjamini and Hochberg; *p < 0.05, **p < 0.01, ***p < 0.001, ****p < 0.0001.

LSEA analysis was performed, using a pre-defined set of classes, subclasses, and common lipid features from the AusDiab cohort, to further explore the associations between lipid remodeling and elevated WC (Fig 3 and S8 Table). Results demonstrated a significant reduction in total ether lipids and subsequent enrichment of diacyl-phospholipids as WC increased, aligning with the lipid ratios (Fig 3). On a class level, 7 of the 8 ether lipid classes measured, including PE(O), PE(P), lyso-alkenyl-phosphatidylethanolamine (LPE(P)), alkyl-phosphatidylcholine (PC(O)), alkenyl-phosphatidylcholine (PC(P)), lyso-alkyl-phosphatidylcholine (LPC(O)) and lyso-alkenyl-phosphatidylcholine (LPC(P)), negatively correlated with WC, while phospholipid classes (PE, phosphatidylinositol (PI), lyso-phosphatidylinositol (LPI), phosphatidylserine (PS) and phosphatidylglycerol (PG)) and neutral lipids (diacylglycerol (DG) and triacylglycerol (TG)) were enriched. Mono-alkyl-diacylglycerol (TG(O)) was the only ether lipid class to show a positive association with WC, though this increase was smaller than that observed for TG species and may be a consequence of the large spike in free fatty acids associated with elevated WC. Similarly, the negative correlation between total PC and WC contrasted with other phospholipid classes, however, this association was not deemed significant (*p-value* <0.05). Lipid features displayed enrichment of saturated and monounsaturated fatty acids involved in the *de novo* lipogenesis (DNL) pathway, while the branched chain fatty acid (BCFA) methylhexadecanoic acid (MHDA) exhibited a negative association with WC (Fig 3). Alternatively, the n-6 and n-3 pathways demonstrated overall positive and negative associations with increasing WC, respectively.

### Transcriptional regulation of ether lipids in obesity corroborates findings from the lipid ratios

**Fig 4:**
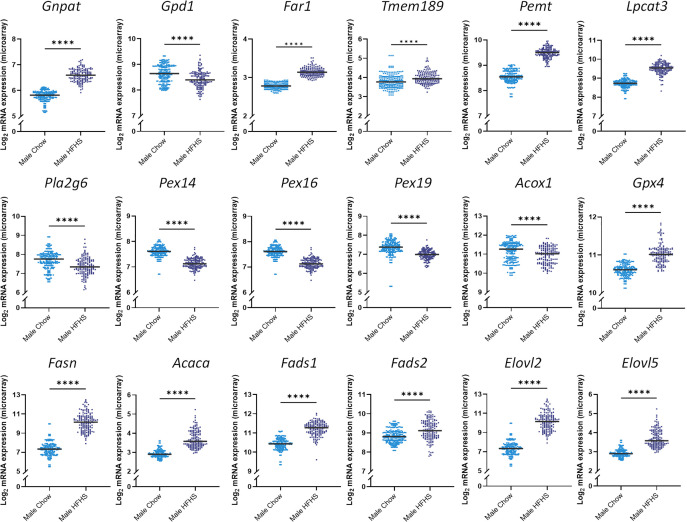
Effect of a high-fat/high-sugar diet on genes involved in ether lipid and fatty acid metabolism. Liver microarray data of male mice across 74 matched strains (*n* = 2–3 per strain) after either a high-fat/high-sugar (navy, *n* = 152) or chow (blue, *n* = 222) diet for eight weeks. Plots depict consolidated micro-array data across each strain. Data was log_2_ transformed and analyzed using Mann–Whitney *U* tests. *Gnpat*: glyceronephosphate-O-acyltransferase; *Gpd1*: glycerol-3-phosphate dehydrogenase 1; *Far1*: fatty-acid reductase 1; *Tmem189*: transmembrane protein 189; *Pemt*: phosphatidylethanolamine N-methyltransferase; *Pex14*: peroxisomal biogenesis factor 14; *Pex16*: peroxisomal biogenesis factor 16; *Pex19*: peroxisomal biogenesis factor 19; *Lpcat3*: lyso-phosphatidylcholine acyltransferase 3; *Pla2g6*: phospholipase A2 group VI; *Acox1*: acyl-coa oxidase 1; *Gpx4*: glutathione peroxidase 4; *Fasn*: fatty acid synthase; *Acaca*: acetyl-Coa carboxylase; *Fads1*: fatty acid desaturase 1; *Fads2*: fatty acid desaturase 2; *Elovl2*: fatty-acid elongase 2; *Elovl5*: fatty-acid elongase 5. Data is publicly accessible from Gene Expression Omnibus under the accession codes: GSE16780, GSE64769; *p < 0.05, **p < 0.01, ***p < 0.001, ****p < 0.0001.

To substantiate the findings derived from the lipid ratios, we utilized liver microarray data from the HMDP. With this dataset, we explored the effects of a HFHS diet, and subsequent obesity, on the expression of genes involved in ether lipid synthesis, with a focus on those highlighted by the ratios. Expression of *Peds1* (encoded by the *Tmem189 gene*) and lyso-phosphatidylcholine acyltransferase 3 (*Lpcat3)* was increased in mice on the HFHS diet for eight weeks compared to the chow diet (Fig 4). In contrast, *iPla2* (encoded by the *Pla2g6 gene*) was decreased, supporting findings from the lipid ratios (Fig 4). Expression of key genes involved in plasmalogen synthesis, including fatty acid reductase 1 (*Far1*), glyceronephosphate-O-acyltransferase (*Gnpat*), and phosphatidylethanolamine N-methyltransferase (*Pemt*), were elevated in the HFHS-fed mice compared to chow-fed controls (Fig 4). Conversely, expression of glycerol-3-phosphate dehydrogenase 1 (*G3pdh*, encoded by the *Gdp1* gene), responsible for the reversible conversion of glycerol-3-phosphate (G3P) into dihydroxyacetone phosphate (DHAP), was reduced in mice on the HFHS diet compared to chow fed controls (Fig 4) [40].

Next, we assessed microarray data of genes involved in fatty-acid synthesis (Fig 4). Mice on the HFHS diet exhibited elevated expression of genes linked to DNL and PUFA synthesis, namely fatty-acid synthase (*Fasn*), acetyl-CoA carboxylase (*Acaca*), *Δ5*- and *Δ6*-fatty acid desaturase (*Fads1*, *Fads2*), and fatty acid elongase 2 and 5 (*Elovl2*, *Elovl5*). Peroxisomal biogenesis factors (Pex), required for the assembly of the peroxisomal membrane and import of peroxisomal membrane proteins, including *Pex14*, *Pex16,* and *Pex19*, were significantly reduced in mice fed the HFHS diet compared to chow-fed mice (Fig 4). Additionally, acyl-coA oxidase 1 (*Acox1*), which resides exclusively within peroxisomes and breaks down very-long-chain fatty acids via β-oxidation, was reduced in response to the HFHS diet for 8 weeks. Glutathione Peroxidase 4 (*Gpx4*) is also localized in peroxisomes and defends against lipid peroxidation. Data from the HMDP demonstrated an increase in *Gpx4* expression in mice on the HFHS diet for 8 weeks compared to chow fed mice (Fig 4).

**Fig 5:**
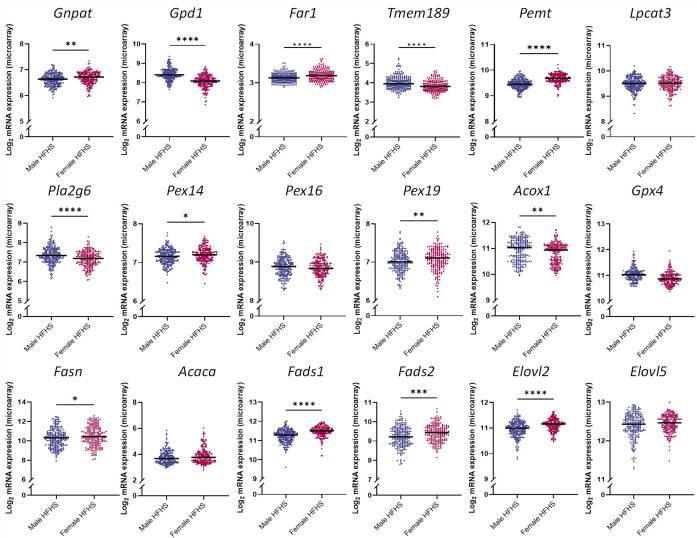
Sex-specific effects of a high-fat/high-sugar diet on genes involved in ether lipid and fatty acid metabolism. Liver microarray data of male (navy, *n* = 227) and female (purple, *n* = 206) mice across 108 strains (*n* = 2-3 per strain) after a high-fat/high-sugar diet for eight weeks. Plots depict consolidated micro-array data across each strain. Data was log_2_ transformed and analyzed using Mann–Whitney *U* tests. *Gnpat*, glyceronephosphate-O-acyltransferase; *Gpd1*: glycerol-3-phosphate dehydrogenase 1; *Far1*: fatty-acid reductase 1; *Tmem189*: transmembrane protein 189; *Pemt*: phosphatidylethanolamine N-methyltransferase; *Pex14*: peroxisomal biogenesis factor 14; *Pex16*: peroxisomal biogenesis factor 16; *Pex19*: peroxisomal biogenesis factor 19; *Lpcat3*: lyso-phosphatidylcholine acyltransferase 3; *Pla2g6*: phospholipase A2 group VI; *Acox1*: acyl-coa oxidase 1; *Gpx4:* glutathione peroxidase 4; *Fasn*: fatty acid synthase; *Acaca:* acetyl-Coa carboxylase; *Fads1*: fatty acid desaturase 1; *Fads2*: fatty acid desaturase 2; *Elovl2*: fatty-acid elongase 2; *Elovl5*: fatty-acid elongase 5. Data is publicly accessible from Gene Expression Omnibus under the accession code: GSE64769; *p < 0.05, **p < 0.01, ***p < 0.001, ****p < 0.0001.

To assess sexual dimorphisms in the expression of genes linked to ether lipid metabolism, we utilized a secondary dataset from the HMDP comprising of male and female mice on a HFHS diet for eight weeks. Results showed elevated expression of genes required for peroxisomal biogenesis and ether lipid synthesis, including *Pex14, Pex19*, *Gnpat*, and *Far1*, in female mice compared to males on the same diet (Fig 5). Additionally, expression of most genes related to fatty acid synthesis (**Fasn, Fads1, Fads2, Elovl2*)* were higher in females compared to males, aligning with results from the interaction analysis (Fig 5). In contrast, male mice exhibited increased transcription of genes localized within the endoplasmic reticulum, namely *Peds1*, *Pla2g6* *and* *Gpd1*.

### Genome-wide association study identifies important regulators of ether lipid metabolism

We integrated the lipid ratios with genomics data from the BHS cohort (*n* = 4,492) to uncover genetic variants associated with changes in ether lipid synthesis. Additionally, we aimed to identify loci that associate with the lipid ratios as a means of validating the pathways and enzymes being represented by them. Genome-wide significant associations can be viewed in S9 Table.

**Fig 6:**
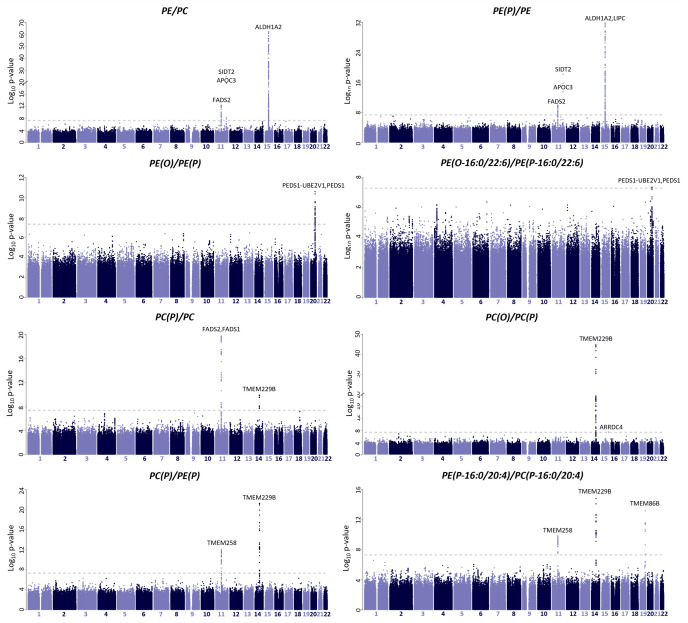
Genome-wide association study on 82 lipid ratios. Genome-wide association analysis (GWAS) was performed on lipid ratios using imputed genotype data from the BHS cohort (*n* = 4,492; 13,887,524 single-nucleotide polymorphisms). Manhattan plots depict loci that associate with the lipid ratio. X-axis shows chromosomal positions, Y-axis shows −log_10_ *p-values,* and gray dotted lines indicate the genome-wide significant threshold (**p-value < 5* × *10**^*−8*^). *ALDH1A2*: aldehyde dehydrogenase 1 family member A2; *LIPC*: hepatic lipase C; *SIDT2*: the systemic RNAi-defective transmembrane family member 2; *APOC3*: apolipoprotein C-III; *PAFAH1B1*: platelet activating factor acetylhydrolase 1b; *CEB164*: centrosomal protein; *FADS1*:fatty acid desaturase 1; *FADS2*: fatty acid desaturase 2; *FADS3*: fatty acid desaturase 3; *TMEM229B*: transmembrane protein 229B; *PEDS1*: plasmanylethanolamine desaturase 1; *CLEC16A*: C-type lectin domain family 16; *TMEM86B*: transmembrane protein 86B; *ARMH4*: Armadillo like helical domain containing 4.

Lipid ratios containing ethanolamine headgroups (PE/PC, PE(O)/PE, PE(P)/PE and LPE/PE) demonstrated significant associations (**p-value* *< 5 × 10^−8^; *p-gain* >820) with loci linked to lipoproteins and PUFA synthesis, namely aldehyde dehydrogenase 1 family member A2 (*ALDH1A2*)/lipase C (*LIPC*) [60 SNPs; top hit: *rs2043085* for the PE/PC ratio; imputation *r*^2^ = 0.991; beta = −0.38; *p-value* = 6.18 × 10^−63^; *p-gain* = 1.43 × 10^+04^], systemic RNAi-defective transmembrane family member 2 (*SIDT2*)/apolipoprotein C-III (*APOC3*) [6 SNPs; top hit: *rs530885291* for the PE(P)/PE ratio; imputation *r*^2^ = 0.794; beta = −2.37; *p-value* = 1.32 × 10^−47^; *p-gain* = 1.47 × 10^+09^] and *FADS1/FADS2/FADS3* [52 SNPs; top hit: *rs99780* for the PC(P)/PC ratio; imputation *r*^2^ = 0.999; beta = 0.21; *p-value = *1.26 × 10^−20^; *p-gain* = 6.38 × 10^+03^] regions (Fig 6 and S9 Table). As these regions are known to be involved in lipid regulation, their associations with the ratios were expected [26]. Alternatively, the PE(O)/PE(P) ratio, which consists of two lipids either side of the *PEDS1* enzyme, exclusively associated with loci containing the *PEDS1-UBE2V1/PEDS1* gene region [96 SNPs; top hit: *rs2664558*; genotyped; beta = −0.16; *p-value* = 2.69 × 10^−10^; *p-gain* = 1.47 × 10^+07^] further validating the linear regression analysis (Fig 6 and S9 Table).

**Fig 7:**
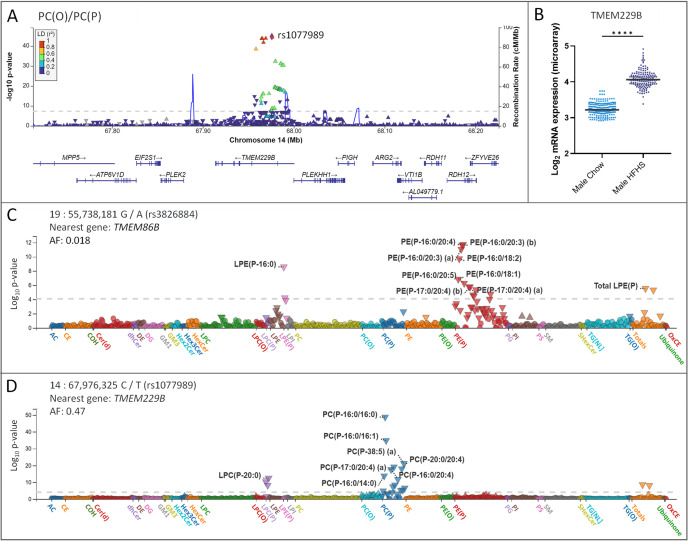
Connecting genomic variations to lipid metabolism. A) Regional association plot of genetic variants against the PC(O)/PC(P) ratio for the transmembrane protein 229B (*TMEM229B)* locus. The single-nucleotide polymorphism (SNP) showing the smallest *p-value* is depicted as a purple diamond and labeled. Other SNPs are color-coded according to the extent of linkage disequilibrium (*r*^*2*^); B) Micro-array data on the *Tmem229b* gene in male mice across 108 strains (*n* = 2–3 per strain) on a high-fat/high-sugar (navy, *n* = 226) or chow (blue, *n* = 254) diet for eight weeks. Microarray data is publicly accessible from Gene Expression Omnibus under the accession code: GSE64769. ****p < 0.0001; C and D) PheWas plots depicting the association between lead variants and lipid species. X-axis: lipid classes, y-axis: −log_*10*_ *p-values*. Each triangle represents an individual lipid species and its orientation depicts the directionality of the association between the lipid species and gene of interest.

As anticipated, lipid ratios capturing PUFA synthesis, namely PE(P-16:0_18:2)/PE(P-16:0_20:4), PE(P-18:0_18:2)/PE(P-18:0_20:4), PE(P-16:0_18:3)/PE(P-16:0_20:5) and PE(P-18:0_18:3)/PE(P-18:0_20:5), exhibited highly significant associations with *FADS1/FADS2/FADS3* loci [575 SNPs; top hit: *rs174564* for the PE(P-18:0/18:2)/PE(P-18:0/20:4); imputation *r*^2^ = 0.999; beta = 0.49; *p-value* = 8.07 × 10^−107^; *p-gain* = 2.60 × 10^+83^]. Interestingly, the PC(P-16:0_20:4)/PE(P-16:0_20:4) ratio, which captures multiple steps in the ether lipid pathway, demonstrated associations with SNPs in the transmembrane 86B (*TMEM86B*) gene region [7 SNPs; top hit: *rs3826884*; genotyped; beta = 0.21; *p-value* = 6.08 × 10^−14^; *p-gain* = 3.08 × 10^+01^] (S9 Table). While these associations did not exhibit a significant *p-gain*, exploring the *rs3826884* variant revealed a strong negative association with many PE(P) [top hit: PE(P-16:0/20:4); beta = −0.20; *p-value* = 1.90 × 10^−12^] and LPE(P) species [top hit: LPE(P-16:0); beta = −0.17; *p-value* = 2.60 × 10^−9^] (Fig 7C).

GWAS results from the PC(O)/PC(P) and PC(P)/PE(P) ratios provided new insights, as they showed highly significant associations with SNPs in the transmembrane protein 229B (*TMEM229B*) gene region [103 SNPs; top hit: *rs1077989* for the PC(O)/PC(P) ratio; genotyped; beta = 0.31; *p-value* = 1.77 × 10^−45^; *p-gain* = 4.31 × 10^+36^] (Fig 7A). Furthermore, the *rs1077989* variant exhibited opposing associations with choline-plasmalogens [Total PC(P), beta = −0.13 and *p-value* = 7.7 × 10^−9^; Total LPC(P), beta = −0.13 and *p-value* = 2.7 × 10^−9^] and choline-ether lipids [Total PC(O), beta = 0.062 and *p-value* = 4.8 × 10^−3^; Total LPC(O), beta = 0.039 and *p-value* = 7.90 × 10^−2^] (Fig 7D). Interestingly, this distinct effect on ether lipid metabolism was consistent with the *rs3826884* variant, however, it demonstrated a specificity for ethanolamine ether lipids rather than choline (Fig 7C).

## Discussion

### Lipid ratios identify potential mechanisms influencing ether lipid metabolism in obesity

Several animal and human studies have reported a negative correlation between ether lipids and acquired obesity [2,15,41,42]. In this study, we further explored this association by generating biologically informed ratios derived from lipid pairs with well-characterized metabolic relationships. By focusing on ratios, rather than absolute concentrations, we aimed to capture more functional aspects of lipid regulation in obesity, such as shifts in precursor or product availability. Linear models revealed strong associations between 82 ratios and obesity-related markers across two datasets, highlighting potential metabolic enzymes and pathway regions affected by obesity. The direction and strength of the association indicated which part of the ratio, either the numerator or the denominator, was more affected by obesity, relative to the other. Results were then integrated with transcriptomic and genomic data to evaluate their capacity to reflect underlying biological relationships. Transcriptomic analyses assessed whether the observed associations aligned to changes in tissue gene expression, while genomic analysis identified loci associated with altered lipid ratios. A key limitation of using regression models with the ratios is that they are inherently associative and cannot provide direct evidence of metabolic flux or alterations in metabolic processors. However, this integrative strategy demonstrates how combining accessible datasets can yield valuable insights into potential mechanisms underlying ether lipid dysregulation in obesity and generate strong leads for further investigation.

Diacyl-phospholipids and ether lipids are both synthesized from G3P, a key glycolysis intermediate involved in multiple metabolic pathways. In the instance of glycerophospholipid metabolism, G3P has two endpoints; it can travel to the endoplasmic reticulum (ER), where it feeds into the Kennedy pathway for diacyl-phospholipid synthesis, or it can be converted into DHAP for ether lipid synthesis. Lipid ratios containing the end products of these two pathways, namely PE(O)/PE, PE(P)/PE, PC(O)/PC, and PC(P)/PC, displayed universal negative associations with WC. This suggests that ether lipid synthesis is reduced relative to diacyl-phospholipid synthesis, as WC increases (Table 1). LSEA analysis supported these results, identifying negative associations between plasmalogen classes and WC, while diacyl-phospholipid classes were positively enriched (Fig 3). This inverse relationship between the two lipid classes and WC aligned with the compensatory mechanisms observed by *Dorninger* and colleagues, whereby PE metabolism adapts to the degree of plasmalogen modulation to maintain a steady state of PUFA content [43]. It could therefore be proposed that the positive association between diacyl-phospholipids and WC reflects an increase in PE metabolism to counteract the known reduction in circulating ether lipids and maintain PUFA levels. Findings from the HMDP suggested that altered hepatic DHAP availability and peroxisomal dysfunction may be additional factors contributing to the reduction in ether lipids relative to diacyl-phospholipids. This was evidenced by a decrease in *G3pdh* expression, which catalyzes the reversible conversion of G3P into DHAP, and markers of peroxisomal biogenesis and function, including *Pex14*, **Pex19*,* and *Acox1*, in mice fed a HFHS diet compared to chow-fed mice (Fig 4). It is worth noting that dietary effects may also be at play in driving the divergence between the pathways. Diets rich in carbohydrates increase blood glucose levels, which is converted into G3P via glycolysis [44].

**Fig 8:**
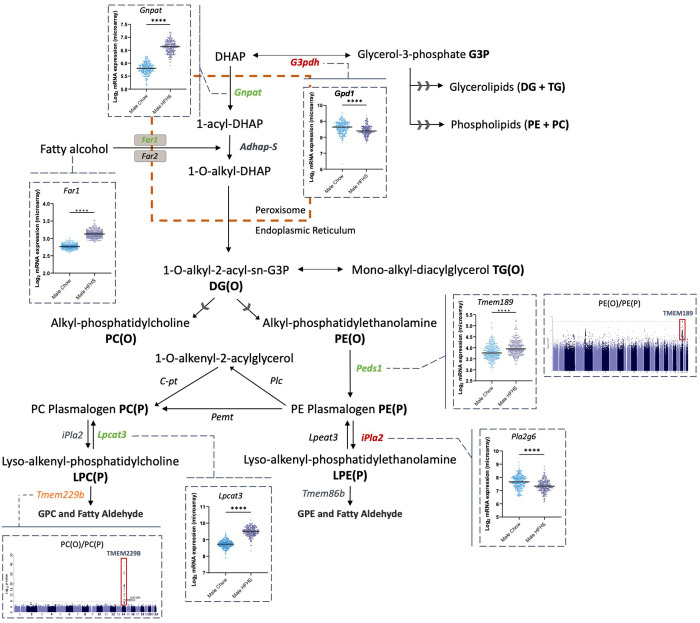
Integration of lipid ratios with multi-omic datasets to explore ether lipid metabolism in obesity. Summary figure details changes in ether lipid metabolism in obesity as indicated by the lipid ratios, liver micro-array data, and GWAS analysis. Coloured text depicts potential changes in enzymatic activity (green: increase; red: decrease), identified through linear regression analysis, adjusting for age and sex, of 82 lipid ratios and waist circumference in the AusDiab cohort (*n* = 10,399). Lipid ratios were log_2_ transformed, mean-centered and scaled to standard deviation. *p-values* were corrected for multiple comparisons using the false discovery rate method of Benjamini and Hochberg. Scatter plots depict consolidated liver micro-array data of male mice across 74 matched strains (*n* = 2−3 per strain) after either a high-fat/high-sugar (navy, *n* = 152) or chow (blue, *n* = 222) diet for eight weeks. Data was log_2_ transformed and analyzed using Mann–Whitney *U* tests; ****p < 0.0001. Manhattan plots show associations between the PE(O)/PE(P) and PC(O)/PC(P) ratios and *TMEM189* and *TMEM229B* loci in the BHS cohort (*n* = 4,492), respectively. Orange text depicts the location of the proposed lyso-plasmalogenase *TMEM229b*. DHAP: dihydroxyacetone phosphate; *Gpd1*: glycerol-3-phosphate dehydrogenase 1 (encodes for G3pdh); *G3pdh*: glycerol-3-phosphate dehydrogenase; DG: diacylglycerol; TG: triacylglycerol; PE: phosphatidylethanolamine; PC: phosphatidylcholine; *Gnpat*: glyceronephosphate O-acyltransferase; *Adhap-S*: alkyl-dihydroxyacetone phosphate synthase; *Far1*: fatty acyl-CoA reductase 1; *Far2*: fatty acyl-CoA reductase 2; *Tmem189*: transmembrane protein 189 (encodes for *Peds1*); *Peds1*: plasmanylethanolamine-desaturase-1; *Plc*: phospholipase C; *Pemt*: phosphatidylethanolamine N-methyltransferase; *C-pt*: choline phosphotransferase; *Pla2g6*: phospholipase A2 group VI (encodes for *iPla2*); *iPla2*: i-phospholipase A2, calcium independent phospholipase A2; *Lpeat3*: lyso-phosphatidylethanolamine acyltransferase 3; *Lpcat3:* lyso-phosphatidylcholine acyltransferase 3*; Tmem86b*: transmembrane protein 86b; *Tmem229B*: transmembrane protein 229b; *GPC*: glycerophosphocholine; *GPE:* glycerophosphoethanolamine.

Alternative lipid ratios, including PE(O)/PE(P) and LPE(P)/PE(P), exhibited more nuanced patterns, as PE(P) species positively associated with WC relative to their upstream precursor and downstream substate (Table 1 and Fig 8). These results allude to the modulation of *PEDS1* and *iPLA2* activity, as these enzymes are solely responsible for converting PE(O) to PE(P) and PE(P) to LPE(P), respectively. If true, this would point to a mechanism that prioritizes plasmalogen synthesis in obesity. Supporting this, GWAS results showed significant associations between the PE(O)/PE(P) ratio and SNPs in the *TMEM189* gene region, which encodes for *PEDS1* (Fig 6). Data from the HMDP also demonstrated the up-regulation of *Tmem189* and down-regulation of *Pla2g6* expression in mice on a HFHS diet compared to controls (Fig 4). Together, these findings allude to a coordinated effort to sustain circulating plasmalogen levels despite their overall negative association with WC. Interestingly, expression of *Gnpat* and *Far1*, the first and rate-limiting enzymes required for ether lipid synthesis, were increased in mice on a HFHS diet compared to a chow diet (Fig 4). While these data did not directly align with the ratios, they may be indicative of a feedback mechanism to compensate for reduced enzymatic activity. However, further mechanistic studies are required to fully elucidate the pathways involved.

### Ratios identify changes in fatty acid synthesis as waist circumference increases

Fatty acids are critical for determining the composition and subsequent function of lipids within a cell. This study used lipid ratios with differing acyl- and alkenyl-chains to explore the association between increasing WC and fatty acid synthesis. Ratios assessing alterations in the alkenyl-chain at the *sn-1* position of plasmalogens, including PE(P-16:0/18:3)/PE(P-18:0/18:3), PE(P-16:0/18:2)/PE(P-18:0/18:2), PE(P-16:0/20:4)/PE(P-18:0/20:4), PE(P-16:0/22:4)/PE(P-18:0/22:4) and PE(P-16:0/22:6)/PE(P-18:0/22:6), demonstrated a positive association between O-16:0 and WC, relative to its O-18:0 substrate (Table 2). As the fatty alcohols present in ether lipids are derived from fatty acid synthase (FAS)-mediated DNL or dietary sources, these results raise the possibility of an increased contribution from the DNL pathway at a higher WC [45]. This is reinforced by the understanding that a surge through the hepatic DNL pathway can contribute to the disease pathology of obesity and fatty liver disease [46–48]. In our study, elevated expression of key enzymes involved in the DNL pathway, namely *Fasn* and *Acaca*, were observed in mice on the HFHS diet in the HMDP, while LSEA analysis showed enrichment of DNL-derived products, including C14:0, C14:1, C16:0 and C16:1, with elevated WC (Fig 3). Although these findings suggest increased activity of the DNL pathway, flux-based studies are required to definitively attribute the observed patterns to changes in DNL. Moreover, gene expression data represents one layer of regulation and does not reflect protein abundance, enzymatic activity or influences from dietary sources. However, this work positions the lipid ratios as potential metabolic readouts of altered fatty acid synthesis in obesity, which may disrupt broader lipid metabolism.

Lipid ratios with differing acyl-chains at the *sn-2* position showed regional associations between WC and various stages of the n-3 and n-6 pathways (Table 2). Specifically, the PE(P-16:0/18:2)/PE(P-16:0/20:4) and PE(P-18:0/18:2)/PE(P-18:0/20:4) ratios demonstrated an inverse relationship with WC, indicating that the abundance of PE(P-16:0/20:4) species increased, relative to PE(P-16:0/18:2) species, with elevated WC. Given that 20:4 is derived from 18:2, this shift in plasmalogen composition signifies a potential change in n-6 metabolism in individuals with greater WC. Data from the HMDP supported this, as expression of primary genes required for PUFA synthesis, namely *Fads1*, **Fads2*,* and *Elovl2*, were elevated in mice on a HFHS diet compared to chow-fed mice (Fig 4). This has also been shown in the literature as high n-6 intake is associated with elevated n-6 metabolism and subsequent obesity [49]. Interestingly, this observation was less pronounced in the corresponding n-3 ratios (PE(P-16:0/18:3)/PE(P-16:0/20:5) and PE(P-18:0/18:3)/PE(P-18:0/20:5)), as the *p-gains* were not significant (Table 2). This disparity between the two pathways, despite their shared enzymatic processes, may reflect the impact of dietary n-3 and n-6 species on endogenous synthesis. Lipid ratios depicting the final stages of PUFA synthesis (PE(P-16:0/22:5) [n3]/PE(P-16:0/22:6), PE(P-18:0/22:5) [n3]/PE(P-18:0/22:6), PE(P-16:0/22:4)/PE(P-16:0/22:5) [n6] and PE(P-18:0/22:4)/PE(P-18:0/22:5) [n6]) exhibited further n-6 effects, as plasmalogen species containing the final product of the n-6 pathway, DPA, were negatively associated with WC, relative to their upstream precursor 22:4 (Table 2). Again, the corresponding n-3 ratios did not exhibit significant *p-gains.* As the final step of DPA synthesis occurs exclusively in the peroxisome via β-oxidation, the reduction in markers of peroxisomal function *(Pex14*, **Pex16*,* and *Pex19)* and β-oxidation (*Acox1)* in the HMDP suggests that this inverse relationship may reflect peroxisomal impairments at a higher WC (Fig 4). Experimental validation is necessary to confirm this hypothesis.

LSEA analysis identified an inverse correlation between MHDA and WC (Fig 3). This observation has been reported before, with groups documenting a reduction in serum and adipose BCFA in obese participants compared to healthy controls [50,51]. Branched-chain-amino-acids (BCAA), specifically leucine, isoleucine and valine, serve as essential substrates for BCFA synthesis, contributing ~25%–30% of the required lipogenic acyl-CoA in adipose tissue [52,53]. Individuals with T2D and obesity have demonstrated negative associations with BCAA catabolism, resulting in elevated levels of circulating BCAA [54–56]. This accumulation creates a flow-on effect, diminishing the availability of branched-chain-acyl-CoA and subsequent BCFA synthesis. This disruption contributes to the dysregulation of genes associated with lipid metabolism and inflammation in adipocytes, further amplifying the disease pathology [57].

### Ratios detect sex-specific differences in ether lipid metabolism as waist circumference increases

In recent years, studies have identified distinct sex differences in lipid synthesis linked to metabolic disease, many of which are driven by hormonal effects [58]. Here, 36 lipid ratios exhibited significantly different associations with WC between sexes, emphasizing disparities in how males and females regulate ether lipid metabolism (Fig 2). Of these ratios, 17 showed significant *p-gains*, though this varied between sexes. These differences imply that one sex had a stronger association with a component of the ratio, thereby overshadowing the ability of the lipid ratio to capture additional nuanced information.

An interesting observation was the presence of DPA-containing species (both n-3 and n-6) in nearly all ratios that showed an opposing association with WC in males and females (PE(P-16:0/20:5)/PE(P-16:0/22:5) [n3], PE(P-16:0/22:5) [n3]/PE(P-16:0/22:6), PE(P-18:0/22:5) [n3]/PE(P-18:0/22:6), PE(18:0/22:5) [n3]/PE(18:0/22:6), LPC(22:5) [sn2]/LPC(22:6) [sn2], PE(18:0_22:4)/PE(18:0/22:5 [n6] and PC(18:0_22:4)/PC(18:0/22:5 [n6]) (Fig 2). This pronounced effect may be attributed to differences in dietary DPA intake between sexes. Alternative ratios containing lyso-plasmalogen species, including LPE(P)/LPE, LPC(P)/PC(P) and LPC(O)/PC(O), showed distinctly different associations with WC between sexes (negative in males and positive in females) (Fig 2). These findings suggest that females have elevated levels of lyso-plasmalogens relative to plasmalogens, with increasing WC, while males exhibit the opposite. Interestingly, these data are at odds with the literature, reporting higher activity of phospholipase A2 (*PLA2*) in males and subsequently elevated levels of lyso-phospholipid species compared to females [58,59]. While *PLA2* isn’t involved in ether lipid metabolism directly, a member of its subfamily, *iPLA2*, is required for the tight regulation of ether lipid metabolism. Supporting this, *Pla2g6* expression was higher in male mice fed a HFHS diet compared to females (Fig 5). Considering that male mice also presented with elevated expression of *Tmem189* compared to females, it is plausible that in males, enhanced plasmalogen synthesis surpasses the rate of degradation. This would result in lower lyso-plasmalogens relative to plasmalogens compared to females, with increasing WC. It’s important to note that the ratios represent relative abundance rather than absolute lipid concentrations. As such, the mechanistic interpretation remains speculative and requires further investigation to confirm.

### GWAS identifies the *TMEM229B* gene as a regulator of choline-ether lipid metabolism

Results from lipid ratios containing PC(P) classes and/or species provided new insights into a regulatory role for *TMEM229B* in choline-ether lipid metabolism. The *TMEM229B* gene is in chromosome 14 at location 14q241. It is relatively ubiquitous, with high protein levels in the skin, thyroid tissues and cerebellum. Several studies have identified lipid-loci associations between phospholipids and the *TMEM229B* gene region in CVD, while others have highlighted a potential involvement of *TMEM229B* in macrophage depolarization [26,60–64]. To date, one study has linked *TMEM229B* to ether lipids, suggesting that it may regulate plasmalogen synthesis and/or degradation by monitoring the subcellular localization of plasmalogen species [65]. However, no final conclusions have been drawn on the functional role of this protein. Here, the leading SNP to correlate with the PC(O)/PC(P) ratio, *rs1077989*, demonstrated positive associations with PC(O) species and negative associations with PC(P) and LPC(P) species (Fig 7C). Given the specificity of these associations, it is possible that *TMEM229B* is involved in regulating PC plasmalogens by monitoring their degradation. Analysis of the PC(P-16:0_20:4)/PE(P-16:0_20:4) ratio built on this hypothesis, as it demonstrated strong associations with both the *TMEM229B* (43 SNPs) and *TMEM86B* (7 SNPs) gene regions (Fig 6). *TMEM86B* is a lyso-plasmalogenase that hydrolyzes the vinyl-ether linkage of lyso-plasmalogens, resulting in a fatty aldehyde and glycerol derivative [66]. Despite the insignificant *p-gain*, the most significant SNP in the *TMEM86B* region for this ratio was *rs3826884*, which showed negative associations with PE(P) and LPE(P) species (Fig 7C). These preliminary findings, generated through the lipid ratios, suggest that *TMEM229B* may mediate endogenous ether lipid levels in a similar manner to *TMEM86B*, with a specificity for choline-ether lipids. Furthermore, *TMEM86B* activity may be selective for LPE(P) species, contrary to existing literature describing its involvement in the catabolism of both LPE(P) and LPC(P). Data from the HMDP further supported these conclusions, as expression of *Tmem229b* was upregulated in mice on the HFHS diet compared to chow-fed mice (Fig 4). Unfortunately, *Tmem86b* was not measured in the liver microarrays from the HMDP, however, alternative studies have reported an increase in the expression of *Tmem86a*, a close homolog of *Tmem86b*, in mice fed a HFD compared to control mice on a normal chow diet [67]. Taken together, these findings provide a novel insight into a potential role for *TMEM229B* in choline-ether lipid catabolism. Additionally, it proposes that while *FAR1* has traditionally been considered the primary feedback mechanism for plasmalogen regulation, the strong influence of *TMEM86B* and *TMEM229B* on the lipid ratios suggests that plasmalogen degradation may play an equally significant role in regulating their levels.

## Limitations

Despite the exciting insights provided by this study, there are some limitations. First, the lipid ratios are surrogate measures for metabolic activity and should be interpreted accordingly. The results derived from the ratios are associative in nature and their functional significance should be examined more directly. The cross-sectional design of the two cohorts limits our ability to establish causal relationships between the lipid ratios and obesity-related markers. The extended storage time of samples before lipidomic analysis should also be noted. The AusDiab plasma samples were stored at −80 °C for 18 years (collected in 2000 and analyzed in 2018), while the BHS serum samples were stored for 22 years (collected in 1994 and analyzed in 2016). It has been demonstrated that lipid profiles remain relatively stable under long-term storage conditions, however, some degree of degradation is unavoidable and should be considered when interpreting the findings [68]. Additionally, the liver microarray data was generated almost 10 years ago, resulting in the absence of important genes involved in ether lipid metabolism, including *Tmem86b* and alkylglycerol monooxygenase (*Agmo*). Moreover, the chow HMDP cohort did not include female mice, limiting our ability to compare diet effects with the female data from the HFHS cohort [30,31]. Given the observed sex-specific associations between lipid ratios and WC, future studies exploring dietary effects on ether lipid metabolism in females would be particularly valuable. Lastly, the sample size of the BHS (*n* = 4,492) poses some constraints on the GWAS analysis. While our group has previously demonstrated sufficient power in the cohort to detect genetic variants affecting lipid species, there is a fundamental limit to the effect size that can be detected [26].

## Conclusions

This study provides compelling evidence that obesity disrupts ether lipid homeostasis at multiple regulatory sites. The use of lipid ratios provided novel insights into ether lipid metabolism not achievable with lipidomics data alone. Specifically, findings showed consistent inverse associations between multiple ether lipid classes and obesity markers, relative to diacyl-phospholipids, as WC increased. Integration with transcriptomic data and LSEA analysis pointed to changes in enzymes involved in DHAP availability and peroxisomal function. The ratios also suggested shifts in fatty acid and PUFA metabolism, as well as sex-specific differences in ether lipid regulation with increasing WC. This study identified a potential commonality between the roles of two transmembrane proteins, *TMEM229B* and *TMEM86B,* in regulating plasmalogen degradation. These findings raise the possibility that plasmalogen catabolism is equally important for the tight regulation of plasmalogen species, alongside the well-characterized feedback loop involving *FAR1*. A key motivation for this study was to demonstrate how this integrative approach offers a powerful framework for investigating complex relationships between lipid metabolism and obesity. Although experimental validation is still required, this study demonstrates the value of integrating large datasets with lipid ratios to uncover strong leads for future, more in-depth investigation.

## Supporting information

S1 Fig Validation of lipid ratios in the Busselton Health Study. Correlation between the regression coefficients of each lipid ratio in the AusDiab cohort (*n* = 10,399; x-axis) and BHS cohort (*n* = 4,492; y-axis). Lipid ratios were log_2_ transformed, mean centered and scaled to standard deviation (SD). WC; waist circumference. Labeled points identify ratios with significant opposing associations with WC between the cohorts.
(TIF)

S1 Table Characteristics of study participants in the Australian Diabetes Obesity and Lifestyle Study and the Busselton Health Study. ^**1**^AusDiab: Australian Diabetes Obesity and Lifestyle Study; ^2^BHS: Busselton Health Study; ^3^HDL-C: high density cholesterol; ^4^FBG: fasting blood glucose; ^5^SBP: systolic blood pressure; ^6^DBP: diastolic blood pressure; ^7^2h-PLG: 2-h post load glucose; ^8^HbA1C: glycated hemoglobin; ^9^HOMA-IR: homeostasis model assessment of insulin resistance. ^a^Values expressed as mean (±SD); ^b^Values expressed as frequency, n (%) for dichotomous variables; ^c^Data in Median, (IQR) as Triglyceride distribution was right skewed.
(XLSX)

S2 Table Multiple reaction monitoring transitions for examined lipid species. ^1^Lipid nomenclature is read as follows: letters denote the head group; () brackets denote the sum composition of the lipid measured including total number of carbons and double bonds; [] brackets denote the product ion monitored on the mass spectrometer. NL: neutral loss; SIM: sum of ions monitored; MRM: multiple reaction monitoring.
(XLSX)

S3 Table Description of the 82 lipid ratios. ^**1**^Lipid nomenclature is read as follows: letters denote the head group; () brackets denote the sum composition of the lipid measured including total number of carbons and double bonds; [] denote the position of the acyl chain; n3 and n6 denote omega-3 and omega-6 poly-unsaturated fatty acid species. *PEDS1*, plasmanylethanolamine desaturase; *PEMT*, phosphatidylethanolamine N-methyltransferase; *C-PT*, choline-phosphotransferase; *PLC*, phospholipase C; *iPLAT*, calcium-independent phospholipase A2; *LPCAT*, lyso-phosphatidylcholine acyltransferase 1; *PLA2*, phospholipase A2; *LCAT*, lecithin-cholesterol acyltransferase.
(XLSX)

S4 Table Associations between 82 lipid ratios and markers of obesity in the Australian Diabetes Obesity and Lifestyle Study. Linear regression analysis, adjusting for age and sex, was performed between 82 lipid ratios and various markers of obesity in the AusDiab cohort (*n* = 10,399). ^1^Lipid nomenclature is read as follows: letters denote the head group; () brackets denote the sum composition of the lipid measured; including total number of carbons and double bonds; [] denote the position of the acyl chain; n3 and n6 denote omega-3 and omega-6 poly-unsaturated fatty acid species; ^2^SD-change denotes the standard deviation change per unit of waist circumference (WC), body-mass-index (BMI) or waist–hip ratio (WHR); ^3^*p-gain* was calculated by dividing the lowest *p-value* among the lipid species used in the ratio by the *p-value* of the lipid ratio. The *p-gain* was deemed significant if it exceeded 10 × the number of ratios tested (*p-gain* >820).
(XLSX)

S5 Table Associations between 82 lipid ratios and markers of obesity in the Busselton Health Study. Linear regression analysis, adjusting for age and sex, was performed between 82 lipid ratios and various markers of obesity in the BHS cohort (*n* = 4,492). ^1^Lipid nomenclature is read as follows: letters denote the head group; () brackets denote the sum composition of the lipid measured including total number of carbons and double bonds; [] denote the position of the acyl chain; n3 and n6 denote omega-3 and omega-6 poly-unsaturated fatty acid species; ^2^SD-change denotes the standard deviation change per unit of waist circumference (WC), body-mass-index (BMI) or waist–hip ratio (WHR); ^3^*p-gain* was calculated by dividing the lowest *p-value* among the lipid species used in the ratio by the *p-value* of the lipid ratio. The *p-gain* was deemed significant if it exceeded 10 × the number of ratios tested (*p-gain* >820).
(XLSX)

S6 Table Interaction analysis between 82 lipid ratios and markers of obesity in the Australian Diabetes Obesity and Lifestyle Study. Linear regression analysis was performed between 82 lipid ratios and various markers of obesity, including sex as the interaction term and adjusting for age, in the AusDiab cohort (*n* = 10,399). ^1^Lipid nomenclature is read as follows: letters denote the head group; () brackets denote the sum composition of the lipid measured including total number of carbons and double bonds; [] denote the position of the acyl chain; n3 and n6 denote omega-3 and omega-6 poly-unsaturated fatty acid species; ^2^SD-change denotes the standard deviation change per unit of waist circumference (WC), body-mass-index (BMI), waist–hip ratio (WHR). ^3^Interaction *p-value* denotes whether the associations between each lipid ratio and obesity-marker are statistically different between males and females. ^4^*p-gain* was calculated by dividing the lowest *p-value* among the lipid species used in the ratio by the *p-value* of the lipid ratio. The *p-gain* was deemed significant if it exceeded 10 × the number of ratios tested (*p-gain* >820).
(XLSX)

S7 Table Interaction analysis between 82 lipid ratios and markers of obesity in the Busselton Health Study. Linear regression analysis was performed between 82 lipid ratios and various markers of obesity, including sex as the interaction term and adjusting for age, in the BHS cohort (*n* = 4,492). ^1^Lipid nomenclature is read as follows: letters denote the head group; () brackets denote the sum composition of the lipid measured including total number of carbons and double bonds; [] denote the position of the acyl chain; n3 and n6 denote omega-3 and omega-6 poly-unsaturated fatty acid species; ^2^SD-change denotes the standard deviation change per unit of waist circumference (WC), body-mass-index (BMI), waist–hip ratio (WHR). ^3^Interaction *p-value* denotes whether the associations between each lipid ratio and obesity-marker are statistically different between males and females. ^4^*p-gain* was calculated by dividing the lowest *p-value* among the lipid species used in the ratio by the *p-value* of the lipid ratio. The *p-gain* was deemed significant if it exceeded 10 × the number of ratios tested (*p-gain* >820).
(XLSX)

S8 Table Lipid set enrichment analysis using plasma lipidomics data from the Australian Diabetes Obesity and Lifestyle Study. Lipidomic analysis was performed on the AusDiab cohort (*n* = 10,399). Enrichment scores were generated by summing the association t-statistics for individual lipids against waist circumference (WC). ^1^Lipid sets denote pre-defined lipid groups used in the analysis. Lipid domains map to lipid species containing the same backbone, i.e., *Sphingolipids*; lipids containing a spingoid base; *Glycerophospholipids*; lipids containing a glycerol base with a phospho-head group; *Neutral lipid*s: sterol lipids and lipids containing a glycerol backbone; lipid subclasses are clustered by unique structural features; lipid classes include all lipids with the respective headgroup; lipid features include any lipid species containing the respective fatty acid at the *sn-1*, *sn-2* or *sn-3* position; ^2^LSEA Score: represents the correlation adjusted sum of the t-statistics for individual lipids against WC. ^3^BH: corrected *p-values* using the false discovery rate method of Benjamini and Hochberg.
(XLSX)

S9 Table Association of genetic variants against lipid ratios in the Busselton Health Study. Genome-Wide Association Study was performed on 82 Lipid Ratios using imputed genotype (13.8 million SNPs) data from the Busselton Health Study (*n* = 4,492). The top 10 SNPs to associate with each lipid ratio were included in the table. ^1^Lipid nomenclature is read as follows: letters denote the head group; () brackets denote the sum composition of the lipid measured including total number of carbons and double bonds; [] denote the position of the acyl chain; n3 and n6 denote omega-3 and omega-6 poly-unsaturated fatty acid species; ^2^SNP: single-nucleotide polymorphism; ^3^EAF: effect allele frequency; ^4^GWAS was performed on lipid ratios and individual lipid species to identify the significance of each SNP; ^5^SE: standard error; ^6^*p-gain* was calculated by dividing the lowest *p-value* among the lipid species used in the ratio by the *p-value* of the lipid ratio. The *p-gain* was deemed significant if it exceeded 10 × the number of significant SNPs identified for each ratio. Significant *p-gains* are depicted in red.
(XLSX)

## Abbreviations

**:**
AusDiab: Australian, Diabetes, Obesity and Lifestyle Study
BCAA: branched-chain-amino-acids
BCFA: branched chain fatty acid
BHS: Busselton Health Study
BMI: body mass index
DHAP: dihydroxyacetone phosphate
dMRM: dynamic scheduled multiple reaction monitoring
DNL: de novo lipogenesis
DPA: docosapentaenoic acid
ER: endoplasmic reticulum
FAS: fatty acid synthase
G3P: glycerol-3-phosphate
GRM: genetic related matrix
HFHS: high-fat/high-sugar
HMDP: hybrid-mouse-diversity panel
iPLA2: independent phospholipase A2
LPAT: lyso-phospholipid acyltransferase
LSEA: lipid set enrichment analysis
MAF: minor allele frequency
MHDA: methylhexadecanoic acid
PQC: pooled quality control
PUFA: polyunsaturated fatty acid
SNPs: single-nucleotide polymorphisms
T2D: type 2 diabetes
WC: waist circumference
WHR: waist–hip ratio
