# Supplementary figures and images for: Alterations in ether lipid metabolism in obesity revealed by systems genomics of multi-omics datasets
Source: PLoS Biol. 2025 Aug 28;23(8):e3003349. doi: 10.1371/journal.pbio.3003349 (PMC12393746; doi:10.1371/journal.pbio.3003349)

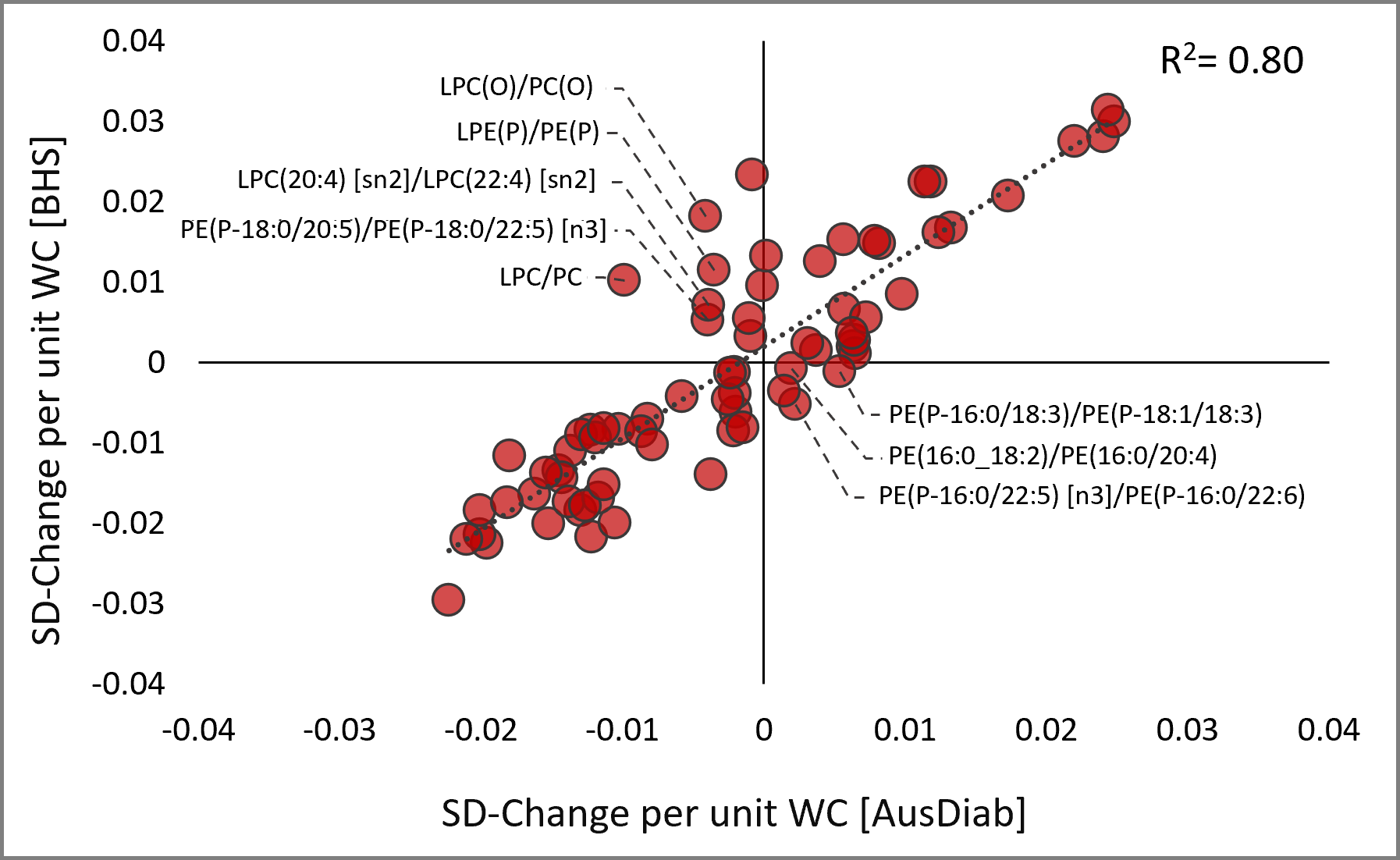

Supplement: S1 Fig — Correlation between the regression coefficients of each lipid ratio in the AusDiab cohort (n = 10,399; x-axis) and BHS cohort (n = 4,492; y-axis). Lipid ratios were log2 transformed, mean centered and scaled to standard deviation (SD). WC; waist circumference. Labeled points identify ratios with significant opposing associations with WC between the cohorts. (TIF) [file pbio.3003349.s001.tif]
